# How do Parents Manage Irritability, Challenging Behaviour, Non-Compliance and Anxiety in Children with Autism Spectrum Disorders? A Meta-Synthesis

**DOI:** 10.1007/s10803-017-3361-4

**Published:** 2017-12-08

**Authors:** Elizabeth O’Nions, Francesca Happé, Kris Evers, Hannah Boonen, Ilse Noens

**Affiliations:** 1 Parenting and Special Education Research Unit, Faculty of Psychology and Educational Sciences, KU Leuven, Leopold Vanderkelenstraat 32, P. O. Box 3765, 3000 Leuven, Belgium; 2 Leuven Autism Research (LAuRes), KU Leuven, Leuven, Belgium; 3 MRC Social, Genetic and Developmental Psychiatry Centre, Institute of Psychiatry, Psychology and Neuroscience, King’s College London, London, UK; 4 Division of Psychology and Language Sciences, Department of Clinical, Educational & Health Psychology, University College London, London, UK; 5 Department of Child Psychiatry UPC, KU Leuven, Leuven, Belgium

**Keywords:** ASD, Irritability, Non-compliance, Challenging behaviour, Anxiety, Parenting strategies, Behaviour management

## Abstract

Although there is increasing research interest in the parenting of children with ASD, at present, little is known about everyday strategies used to manage problem behaviour. We conducted a meta-synthesis to explore what strategies parents use to manage irritability, non-compliance, challenging behaviour and anxiety in their children with ASD. Approaches included: (1) accommodating the child; (2) modifying the environment; (3) providing structure, routine and occupation; (4) supervision and monitoring; (5) managing non-compliance with everyday tasks; (6) responding to problem behaviour; (7) managing distress; (8) maintaining safety and (9) analysing and planning. Results suggest complex parenting demands in children with ASD and problem behaviour. Findings will inform the development of a new measure to quantify parenting strategies relevant to ASD.

**Electronic supplementary material:**

The online version of this article (doi:10.1007/s10803-017-3361-4) contains supplementary material, which is available to authorized users.

## Introduction

Autism spectrum disorders (ASD) are neurodevelopmental impairments characterised by difficulties with communication, socialisation, and rigid and repetitive behaviours (American Psychiatric Association 2013). Although not part of the diagnostic criteria, problem behaviour is very common in ASD, and is more severe in ASD compared to typical development or in the context of intellectual disability (e.g., Blacher and McIntyre 2006; Brereton et al. 2006; Eisenhower et al. 2005; Estes et al. 2009).

Problem behaviour in ASD includes particularly troublesome features, such as self-injury, running away, aggression, property damage, and inappropriate behaviour in public (often termed “challenging behaviour”). Extreme irritability (e.g., anger, frustration, distress, meltdowns), and persistent non-compliance with everyday demands also present considerable challenges. These behaviours have been identified as important treatment targets in children with ASD (e.g., McGuire et al. 2016; Chowdhury et al. 2010). Subjectively, parents report experiencing behaviours that necessitate constant supervision, make the child stand out from others, and provoke others’ embarrassment or annoyance as particularly problematic (Turnbull and Ruef 1996).

In addition, approximately 30–42% of youth with ASD meet criteria for an anxiety disorder (Simonoff et al. 2008; van Steensel et al. 2011; White et al. 2009). There has been debate as to whether anxiety represents a co-occurring feature in ASD, or whether it arises as a result of cognitive factors, or as a downstream consequence of problematic interactions with the environment (e.g., Bearss et al. 2016). Parents reportedly attribute problem behaviour associated with stressors (e.g., changes in the daily routine, feared stimuli) to anxiety, due to the child’s arousal, distress, and attempts to escape (e.g., Bearss et al. 2016). Anxiety-related avoidance has also been reported in response to routine requests in children with ASD (e.g., Lucyshyn et al. 2004), evidenced by escape behaviours and arousal when demands are pursued. Escape-driven avoidance appears to promote the development of coercive cycles of parent–child interaction surrounding daily activities, and the progressive erosion of family routines (Lucyshyn et al. 2004).

### Problem Behaviour in the Family Context

Problematic responses to everyday situations in children with ASD are thought to represent an interaction between child cognitive factors and triggers in the environment to which the child is sensitive. Child factors include poor social awareness/abnormalities in social-information processing (e.g., Dominick et al. 2007); heightened arousal or emotional dysregulation (e.g., Bearss et al. 2016; Mazefsky et al. 2016); rigidity (e.g., Marquenie et al. 2011); intolerance of uncertainty (e.g., Rodgers et al. 2012); and sensory sensitivities (e.g., Schaaf et al. 2011). Vulnerabilities manifest in triggering environmental contexts, e.g., when feared stimuli are present (e.g., Neufeld et al. 2014), in the context of routine demands (Lucyshyn et al. 2004, 2015); in the absence of parental attention/provision of specific activities (Marquenie et al. 2011; Lucyshyn et al. 2004, 2007); when there are changes in routines/environments (e.g., Ludlow et al. 2012); or when things are not ‘on the child’s terms’ (e.g., Larson 2006; DeGrace 2004).

At present, relatively little is known about how parents manage problem behaviour in the context of ASD. Convergent evidence suggests that parents of children with ASD adjust their parenting strategies. Mothers parenting children with ASD reportedly spend 50% more time with their children compared to parents of typical children (9.7 waking hours per day on average, compared to 6.1 h) (Tunali and Power 2002). Qualitative studies report numerous adaptations made by parents to scaffold the child’s inclusion in activities, provide occupation and support the completion of routine tasks (e.g., dressing, grooming, bathing) (e.g., Larson 2006; Schaaf et al. 2011). Parents also make more effort to stimulate their child’s development compared to parents of typically developing children (Lambrechts et al. 2011; Maljaars et al. 2014).

The burden of managing children with chronically high needs has a considerable impact on family members (Gray 1997). The severity of the child’s problem behaviour in the context of ASD plays a major role in the severity of stress that parents experience (Davis and Carter 2008; Estes et al. 2009; Hastings et al. 2005; Lloyd and Hastings 2007). Parenting stress and problem behaviour also appears to be under reciprocal influence (Lecavalier et al. 2006). This is not surprising, given that overwhelmed parents are less likely to be able to respond adaptively to very extreme forms of problem behaviour.

Considerable research exists on interventions to train parents to manage problem behaviour in ASD. Parenting interventions typically aim to increase parental knowledge, skills and confidence in managing problem behaviour, by helping parents to identify triggers, foster more positive interactions, and address maintaining factors (e.g., Hodgetts et al. 2013a, b). However, they vary considerably in philosophy, and consequentially in the approach recommended. For example, whilst some advocate positive approaches and target pre-emptive management (e.g., Lucyshyn et al. 2015), others incorporate consequences (e.g., time out) to dis-incentivise problem behaviour (e.g., Agazzi et al. 2013; Armstrong and Kimonis 2013; Armstrong et al. 2015). Still others aim to foster parental psychological resources rather than teaching a particular management approach (e.g., Singh et al. 2007).

Strikingly however, beyond qualitative reports and case studies, little is known about what parents really do in practice. The aim of this study is to capitalise on the extant literature from qualitative, observational and case studies to identify how parents and caregivers spontaneously manage problem behaviour in ASD. A synthesis of reports and observations on this topic is necessary to provide a rigorous platform to guide further investigation, and inform the development of a new measure to quantify specific parenting strategies relevant to the management of problem behaviour in ASD.

This study uses a recently developed analytic approach termed ‘meta-synthesis’ to address this question (Thomas and Harden 2008; Atkins et al. 2008). This approach, derived from meta-ethnographic research (Doyle 2003), makes it possible to synergise findings from a range of sources, including qualitative studies and other empirical literature. It is increasingly being applied to a range of medical and applied research questions (e.g., Sibeoni et al. 2017; Daker-White et al. 2015). First, a systematic literature search is conducted to identify relevant studies containing information pertinent to the research question. Thematic analysis is conducted to inductively generate descriptive themes and summarise findings across studies (Thomas and Harden 2008). Descriptive themes are then organised into higher level concepts that best address the questions that motivated the synthesis.

Given the exploratory nature of this work, we make no specific predictions about our findings. Descriptive themes will be used to inform the development of question items to measure specific parenting strategies related to managing problem behaviour in ASD.

## Methods

The study procedure involved six stages: (1) definition of the scope of the synthesis; (2) systematic search and identification of relevant papers; (3) extraction of relevant material from papers; (4) annotation of identified exemplars of parenting strategies; (5) development of descriptive themes; (6) critical appraisal and synthesis.

### Definition of the Scope of the Synthesis

#### Parenting Strategies

In line with prior work (e.g., Lambrechts et al. 2011; Maljaars et al. 2014), we conceptualised parenting strategies as concrete, specific, observable behaviours that parents engage in when interacting with their child. This included behaviours that were goal oriented (aiming to prevent problem behaviour), and response oriented (reactions to problem behaviour). We extended the scope to incorporate parental accommodations that had an indirect impact on child behaviour (e.g., advance preparation or planning). This was motivated by the relevance of these strategies to particular goals regarding the management of child problem behaviours.

#### Child Problem Behaviours

Our focus was on dimensions of child problem behaviour of particular theoretical and practical interest: challenging behaviour, irritability, non-compliance or avoidance of demands, and anxiety. This motivated the selection of search terms for our database search. Child problem-behaviours that were the target of parental intervention in our extracted exemplars ranged in severity from mild instances of non-compliance (e.g., non-completion of homework, breaking rules in games), to severe challenging behaviour (e.g., physical attack, running off, self-injury).

### Systematic Search and Identification of Relevant Papers

The systematic search involved two screening phases. The first phase assessed studies identified against the inclusion criteria provided in Table 1. This yielded studies reporting parenting strategies in the context of child problem behaviours.

**Table 1:** Inclusion criteria for Phase 1 screening

Inclusion criteria for Phase 1 screening	
(a)	English language peer-reviewed journal articles, reporting empirical findings or case reports/descriptions/series
(b)	Reporting on parenting strategies with the goal of managing the following dimensions of child problem behaviour: irritability, non-compliance or demand avoidance, challenging behaviour and anxiety
(c)	The majority of children reported to have an autism spectrum disorder^a^; or data for individuals/groups with an autism spectrum disorder can be reviewed separately from other individuals/participant groups
(d)	Includes reports on management of behaviour in children aged under 18 years (i.e. not an exclusively adult sample)

^a^Autism spectrum disorder includes autism, Asperger’s syndrome, atypical autism or pervasive developmental disorder—not otherwise specified

To identify relevant studies, a targeted search strategy was implemented, involving (1) database searches (PsychINFO and Web of Science), (2) a review of studies citing or cited by those identified for inclusion on the basis of the database search, and (3) a review of articles included in two relevant existing meta-syntheses (one on parenting children with autism, the other on parents’ experiences of caring for children with ASD; Ooi et al. 2016; DePape and Lindsay 2015). This approach was chosen because indexing of parenting strategies in response to problem behaviour in ASD within databases was poor. Therefore, database searching alone was not considered to be the most efficient way to find studies containing relevant material.

The systematic search of PsychINFO and Web of Science databases included studies available from database inception until the 19th December 2016. A combination of search terms was used, including autis*—ASD—ASC—pervasive developmental disorder*—PDD* AND anxiet*—irritab*—noncompl*—non-compl*—demand avoid*—avoids demand*—challenging* AND parent* AND behav*. We made the decision not to include other related terms with wider usage outside of ASD (e.g., oppositional, disruptive), because doing so would have resulted in an unfeasibly large number of records to review. Notably, the aim of a literature search conducted as part of a meta-synthesis is not to identify all possible relevant records, but rather to identify sufficient records to produce thematic saturation during the analysis phase.

**Fig. 1:**
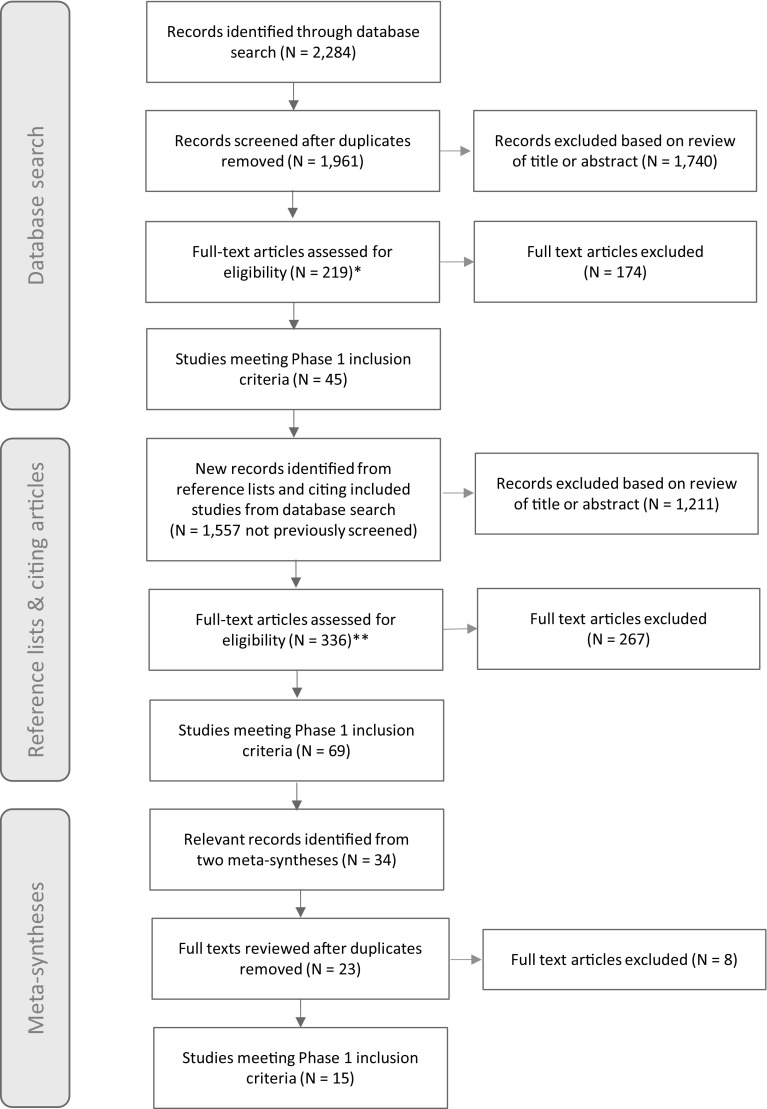
PRISMA flow-chart describing the phases of study identification (Moher et al. 2009). Notes: *Full-texts for two potentially relevant studies could not be retrieved; **Full texts for ten potentially relevant studies could not be retrieved

The database search initially yielded 2284 papers (Fig. 1). Duplicates were excluded, leaving 1961 papers. Of these, 1740 were excluded on the basis of the title or abstract content as they were not relevant, and 219 papers were reviewed in full (full texts for two potentially relevant articles could not be retrieved). A total of 174 studies were subsequently excluded because they did not meet the inclusion criteria for in-depth screening (e.g., they did not contain reports of parenting strategies; they did not have the specific focus of non-compliance, irritability, anxiety and challenging behaviour in ASD; they reported only fidelity to specific trained intervention procedures; they did not describe an empirical study or case description).

Next, the reference lists of the 45 articles meeting inclusion criteria identified in the database search were “back searched” for additional references, and Google Scholar’s “cited by” feature was used to “forward search” for articles citing them. Reference list and citation searching was conducted during January and February 2017. This search revealed 1557 records that had not been previously screened. Of these, 1211 were excluded on the basis of the title or abstract content as they were not relevant. In total, 336 papers were reviewed in full (full texts for 10 potentially relevant articles could not be retrieved). Two hundred and sixty-seven articles were subsequently excluded, leaving an additional 69 studies meeting criteria for in-depth screening.

Finally, references were identified for full-text review from two published meta-syntheses, retrieved during the “forward search” of included articles (Ooi et al. 2016; DePape and Lindsay 2015). This revealed 34 potentially relevant studies, of which 23 had not previously been reviewed. Fifteen of the twenty-three articles met the inclusion criteria for in-depth screening.

The second screening phase assessed whether the 129 studies identified in Phase 1 contained exemplars of naturalistic parenting strategies in response to child problem behaviour, either reported directly (e.g., quotes), or presented descriptively as part of a case description, qualitative or observational report (i.e. researcher-identified themes). In order to meet this criterion, studies had to have collected semi-structured interview data, open-ended parental reports (e.g., open response text), or have reported descriptively on observations or parent reports of parent–child interactions (including case studies/ case descriptions). Studies that only reported clinician- or researcher- defined parenting strategies (e.g., parental directiveness, intrusiveness, warmth etc., measured using questionnaires or structured observations with no additional descriptions) were excluded. Of the N = 129 studies, 56 were dropped. This left a final sample of 73 studies, of which n = 15 were case studies or descriptions, n = 8 were case series, and n = 50 studies reporting qualitative data or thematic analysis.

### Extraction of Relevant Material

Developing our synthesis first involved a careful review of included articles and the extraction of “first order exemplars” (i.e. direct quotes from parents), and “second order exemplars” (i.e. parent strategies presented descriptively as part of a case description, qualitative or observational report). Exemplars were identified from “Results” and “Discussion” sections, and extracted in full into Microsoft Excel spreadsheets by EO. Content extracted included both the goal of the strategy with respect to the child’s behaviour (where explicitly stated) and the parenting strategy.

In some cases, parents reported on strategies implicitly reflecting (rather than actually describing) preventative measures to avoid problem behaviour in their child (e.g., ‘we have to monitor him 24 h a day’). Where these reports were in the context of a study describing child problem behaviour in our domains of interest, they were retained in the analysis. However, if the study did not provide further contextualising detail describing child problem behaviour, it was excluded. Studies describing behavioural interventions were included if pre-intervention (i.e. naturalistic) parenting strategies were reported, and only descriptions of pre-intervention parenting strategies were extracted for the analysis.

To address the aims of our synthesis, we were concerned with the detail of the reported data, rather than the rigour or conceptual development offered by any analyses. Indeed, only a minority of studies explicitly considered parent behaviours to manage problem behaviour in terms of reported thematic constructs or conclusions. Therefore, we did not include, exclude or weight articles based on an appraisal of their quality.

### Annotation of Identified Exemplars

The first stage of analysis involved annotating exemplars to capture meaning and key content (similar to line-by-line coding; Thomas and Harden 2008). Separate annotations were made to describe the goal of parenting strategy with respect to child behaviour (implicitly or explicitly stated), and the parenting strategy itself. For second order exemplars (i.e. those already in the form of a summary), annotations were drawn directly from the exemplar. Annotations were initially made by one member of the research team (EO for qualitative studies; HB for case studies/series), and then reviewed and extended by a second (KE for qualitative studies; EO for case studies/series). For qualitative studies, 87% of annotations relevant to parenting strategies came from EO, and the 13% from KE. For case studies/series, 65% came from HB, and 35% from EO.

### Development of Descriptive Themes

The second stage of the analysis involved organising annotations from different studies into related areas to construct descriptive themes. This involved an iterative process in which members of the team repeatedly labelled and re-organised exemplars into descriptive themes until they achieved consensus. A total of 45 descriptive themes identified in the analysis are presented here. All but one study (Shaked 2005) provided exemplars that fit within descriptive themes.

### Critical Appraisal and Synthesis

The final analytic stage involved organising descriptive themes into broader concepts best able to address the question of how parents manage problem behaviour in ASD. This required the generation of concepts to summarise our findings. The thematic structure developed over discussions amongst the research team, and was modified until consensus was achieved. Nine higher-order concepts were identified, incorporating all descriptive themes.

#### Overview of Included Studies

##### Case Studies/Descriptions

A total of 15 case studies or descriptions were identified, presenting information about 15 children reported to have an ASD diagnosis, and 28 parents, 15 of whom were mothers. The mean age of children was 6.53 years (range 3–13). Eleven out of 15 children were male. Nine were reported to have an intellectual disability, to be non-verbal or have limited speech (data unavailable for two studies). All but one of the child participants was reported to have problem behaviours that were our particular focus (e.g., aggression, non-compliance, tantrums, anxiety). The remaining study reported parent-identified non-compliance with rules during games. Thirteen of the case studies reported on families in the US, one reported on a family in Canada, and one on a family in Turkey. Further details are provided in Supplementary Table 1.

##### Case Series

A total of eight case series were identified, presenting information relating to 37 children, and 39 parents (N not reported for one study). The pooled average age of child participants was 7.83 years [range 2–15 (for n = 3 studies, mean of range used to estimate mean)]. Twenty-two out of the 27 child participants for whom gender information was available were male (data unavailable for one study), and 14 out of 21 were reported to have an intellectual disability, to be non-verbal/have limited speech, or to have cognitive delays (data unavailable for three studies). All but one case series reported that participants were selected due to problem behaviours that were our particular focus (e.g., aggression, non-compliance, tantrums, anxiety). Three of the eight studies included children not reported to have an ASD diagnosis (n = 4 children). These studies were retained in the analysis, and only exemplars pertaining to children with ASD extracted. Six of the case series reported on families in US, one reported on families in the UK, and one reported on families from South Korea. Full details are provided in Supplementary Table 2.

##### Qualitative Studies

A total of 50 studies reporting qualitative data or thematic analysis of qualitative findings were included. The total number of parent participants across studies was 1536 (N per study: range 4–493, median = 14, information not reported for N = 1 study). Parents reported on 1207 children (N not reported for N = 10 studies). The mean age was 9.7 years (range 1–57, data unavailable for 18 studies). At least 17 studies included child participants aged over 18 years (data on range unavailable for four studies). On average, 82% of children were male (data unavailable for 21 studies), and 56% percent of children were reported to have an intellectual disability, to have been diagnosed with autism rather than Asperger syndrome or PDD-NOS, to be cognitively delayed, or to be non-verbal/have limited speech (data unavailable for 28 studies). Four studies included a minority of children without an ASD diagnosis, all of whom presented similar challenges to the ASD sample. Six studies had identified study participants due to problem behaviours (e.g., aggression, non-compliance, tantrums, anxiety). Twenty studies were based in the US, seven in Canada, eleven in Australia, six in the UK, two in Turkey, one in Singapore, one in China, one in Israel, and one in India. For 23 out of the 25 studies that reported information on participant ethnicity, the majority of families were Caucasian. Full details are provided in Supplementary Table 3.

## Results

Descriptive themes derived from the anlaysis, organised into broader conceptual themes, are presented below, with illustrative exemplars drawn from studies. Themes are summarised in Supplementary Table 4, and references provided for studies reporting exemplars associated with each sub-theme.

### Accommodating the Child

Parents reported adapting routines to accommodate the child by following the child’s ‘unique rules’ for how things should be done (e.g., Ausderau and Juarez 2013). This involved accepting the child’s preference for sameness (e.g., providing the same meal each night; Bagatell 2015), and following precise sequences of actions: “He [Bob] has a particular round pillow, and then he has another pillow, and then he has his blankets up and I put the pillow over the top. But before he snuggles in, I have to have a drink of water ready to go because if I don’t he screams out for water when I am gone. So he has the smallest sip of water... and then he lies down and if I don’t have the pillow there he screams for that too.” (Marquenie et al. 2011, p. 151). Parents avoided doing things that the child disliked, which were likely to provoke problem behaviour (e.g., not talking to their older daughter when the younger daughter with a disability was present; Lucyshyn et al. 2004).

Parents planned activities to accommodate the child to reduce the risk of problem behaviour (e.g., Farrugia 2009; Fletcher et al. 2012). This included doing things at the time of day when the child functioned best, attending events when they were less crowded (Fletcher et al. 2012; Schaaf et al. 2011), and locating activities of interest to the child in advance of outings (e.g., Larson 2006).

Parents reported adjusting expectations depending on the child’s mood (e.g., Larson 2006; Foo et al. 2015). For example, after a day at school, parents reduced demands to allow for the fact that the child’s energy levels were depleted (Fletcher et al. 2012). Parents also adapted behavioural goals so that they were achievable (Gray 2003), and gave extra time to complete tasks (Safe et al. 2012). They took cues from the child when deciding whether to pursue an activity: “[We will] get up in the morning. See what he’s like when we get up and then we’ll make plans of what we’re going to do.” (Gray 2003, p. 638). They also took the child home early if they seemed to be finding it hard to cope during outings (e.g., Fletcher et al. 2012).

Parents reported setting priorities and picking battles when deciding whether to insist on things to manage the risk of outbursts and difficult behaviour (e.g., Safe et al. 2012; Larson 2006; Farrugia 2009; Marquenie et al. 2011). They also gave latitude with regards to rules and expectations that applied to other family members, e.g., excusing the child from sitting at the table to eat with the family, or allowing the child to withdraw or engage in repetitive calming behaviours in public (Ausderau and Juarez 2013; Bagatell 2015; Marquenie et al. 2011; Larson 2006).

Parents reported reducing demands when problem behaviour occurred (e.g., Ausderau and Juarez 2013; Marquenie et al. 2011): “Three parents independently stated that attempting to enforce routine related demands on their child by not backing off in the face of problem behavior would only exasperate stress levels in the home and might lead to the break-up of the family (e.g., seeking out-of-home placement for the child).” (Lucyshyn et al. 2004, p. 15). Parents also gave in to the child’s demands for activities or attention to reduce the likelihood of extreme disruption (e.g., Lucyshyn et al. 2004; Divan et al. 2012; DeGrace 2004; Koydemir-Özden and Tosun 2010; Pengelly et al. 2009): “If the child wants to get into the car and have a ride, you must do it, otherwise he gets ill-tempered, yells at midnight, you won’t believe it, one night I had to drive him around from 01 till 04 in the morning, then I slept for 2–3 h and went to work.” (Aylaz et al. 2012, p. 399).

### Modifying the Environment

Parents reported making an effort to limit their child’s exposure to sensory stimuli that they found aversive. This included avoiding using noisy appliances when the child was present and minimising exposure to problematic food items (e.g., Schaaf et al. 2011, Dickie et al.^2009^; Duignan and Connell 2015). Parents also attempted to avoid situations (e.g., activities, events, places) that the child found difficult: “There are things that you say to yourself like this is too big, this room, there are too many people here, it’s too loud, we gotta go.” (Schaaf et al. 2011, p. 383). In particular, parents avoided events that were over-stimulating, or which involved feared stimuli (e.g., Bagatell 2015; Schaaf et al. 2011; Neufeld et al. 2014). They also avoided new or different environments (Mount and Dillon 2014).

Parents reported limiting social activities and outings with the child (e.g., shopping, visiting restaurants; Divan et al. 2012; Gray 2003; Myers et al. 2009, p. 680). They avoided taking the child to friend’s houses or family events, or strictly controlled the time they spent there: “It’s 1 h max; otherwise, it can be a disaster.” (Bagatell 2015, p. 55–56). Frequently, the child’s difficulty tolerating novel environments resulted in one parent staying at home with the child (e.g., Preece and Almond 2008), reducing the risk of an outburst or behaviour that others would find annoying or distressing. Avoiding outings altogether accommodated the child’s preference for sameness: “We stayed home most of yesterday. We couldn’t go out anyway because it’s his routine. Couldn’t leave the house” (Hodgetts et al. 2013a, p. 2579).

### Providing Structure, Routine and Familiarity

Parents reported sticking to fixed routines to manage daily activities (e.g., mealtimes, bedtimes, bathing, dressing etc.; Larson 2006). This reduced the likelihood of the child encountering novel or unexpected stimuli, and thus the risk of an outburst (Schaaf et al. 2011). Routines were also used to help the child transition from one activity to another (Kuhaneck et al. 2010). Families were motivated to stick to routines to reduce the risk of problem behaviour: “His father...hadn’t poured his glass of milk yet [for breakfast] and Nathan just decided that [his dad] had ruined his whole day. … He [didn’t understand that his dad] doesn’t know automatically that he needs a glass of milk…You have to ask [Nathan] if he wants a glass of milk and let him say “yes.” Because if you don’t ask him, then he gets mad when you give it to him…It’s like a dance.” (Larson 2006, p. 73).

Parents also reported providing structure and occupation for their child at all times, in particular during “empty” time (e.g., Turnbull and Ruef 1996); “[…] the days that are the hardest are like Monday, the public holiday, because it was raining and we really had to work hard… to keep him occupied” (Duignan and Connell 2015, p. 203–204).

Parents used picture schedules or lists to inform the child about upcoming activities, so that they knew exactly what to expect: “Being able to talk your child through the steps, like you said, through a white board or, for my child who’s nonverbal, being able to write it out for him so he can see exactly what’s going to happen, we’re going to have to drink something before we can leave, we need to do this.’’ (Johnson et al. 2014, p. 389). Schedules could also reduce anxiety in non-routine situations (e.g., in a hospital setting): “[…] she [the nurse] learned that this child was high functioning and cognitively aware of his pain and the management of it, but obsessed with wanting to control his pain medications. With the father’s input, the nurse developed a schedule of times for medication administration, nursing procedures, meals, and any lab or procedural studies being planned for the next 24 h.” (Scarpinato et al. 2010, p. 252). Visual reminders were also used by parents to facilitate performance of daily routines and transitions between activities (e.g., Clarke et al. 1999; Fettig et al. 2015).

Parents informed the child in advance about any changes in routine: “There has to be a long conversation about numerous plans. She has to have routine and scheduling; we have a calendar... but mostly she prefers to repeatedly verbalise what her arrangements are until you all want to scream” (Duignan and Connell 2015, p. 204). Parents prepared their child for events by giving details in advance (e.g., Johnson et al. 2014; Bearss et al. 2016): “Even to get his hair cut, I have to tell him a week ahead… last night I said, “Okay, there are going to be six people here and there’s going to be two kids”. And I said, “All you have to do is say hello to them and then you can go wherever you want to go”” (Gray 1997, p. 1101). This sometimes involved using “social stories” or scripts to model the steps of an activity, or showing the child pictures of new people or places to increase familiarity (e.g., Ludlow et al. 2012; Johnson et al. 2014). Advance notice was essential in helping the child tolerate new things: “[…] if we have to, on the spot, break it to him that, no, there’s somebody else you have to see, every point is a trigger point for a massive meltdown’’ (Johnson et al. 2014, p. 389).

Parents attempted to keep things as predictable and familiar as possible e.g., avoiding having visitors in the home when the child was around (e.g., Ludlow et al. 2012; Larson 2006), and giving adequate warning before transitions (Kuhaneck et al. 2010; Safe et al. 2012). Familiarity was perceived as helpful reducing problem behaviour: “He tends to do best with well-practiced activities and activities that he does within the context of a structure and with people and an environment that he’s familiar with […] when it’s not a practiced skill or he’s not given that structure, and/or he doesn’t understand what’s going on, then we will see a spike in sort of negative behavioral issues.” (Johnson et al. 2014, p. 389).

Parents reported that introducing new things gradually helped the child to cope: “When he tries something new, the first time he is going to be really mad, the second time he is going to be a little pissed but he is going to do it, and the third time he does it, it is fine.” (Stoner et al. 2007, p. 30). Introducing things step-by-step, e.g., visiting places ‘just to look’ before any demands were placed on the child (Stoner et al. 2007) gradually built up their tolerance, which in turn increased their repertoire of activities: “Two years ago, we couldn’t go to the beach... [He screamed] like you were skinning him...he couldn’t deal with the sensory things, the sand, the sun, the noise of the waves. He would literally hide under a blanket… So we would have to take him home… I knew it was torture for him, but [gradually we’d go back] for just a half hour [and then] go home… Now he loves the beach... we can go to the beach.” (Larson 2006, p. 76).

### Supervision and Monitoring

Parents reported needing to supervise their child at all times (e.g., Zhou and Yi 2014; Fong et al. 1993; Myers et al. 2009): “If we’re lucky, he gets up at 6, and if we’re not you get up at 3, 4… And the minute he’s up, you’re on… Keep him in [the house]. Try to feed him … make sure he’s entertaining himself in a quieter way” (Larson 2010, p. 20). Supervision was required to ensure timely parental intervention, particularly when out in the community: “Someone needs to be monitoring his behavior at all times. …What’s going to make another mom say ‘Get your rotten kid away from my kid!’? You kind of have to gauge that.” (Schaaf et al. 2011, p. 383). Supervision was also necessary during routines to compensate for difficulties remaining on task (e.g., Larson 2010).

Parents described needing to stay alert and ready to intervene: “You always have to be there. To avoid damage, you have to grab him, and you have to be super fast.” (Hodgetts et al. 2013b, p. 169). Vigilance was particularly important in potentially uncertain situations, such as when out in the community or interacting with others (e.g., Fairthorne et al. 2014), to mitigate the risk of problems: “We go to family get-togethers, and their kids just run around, the parents are drinking wine and not paying attention, and the two of us, they’re like, ‘you need to relax.’ We can’t. We literally have to be there around our kids for safety.” (Hodgetts et al. 2013b, p. 169). Vigilance was also required in potentially dangerous everyday situations (e.g., when the child had access to cutlery, or when travelling by car, Bourke-Taylor et al. 2010).

Indicators of the child’s stress and emotional state were a particular target of parental vigilance (e.g., Larson 2010). Parents reported making an effort to keep the child’s mood stable (e.g., Sabapathy et al. 2016; Larson 2010): “Well, it is almost like a home with an alcoholic. You walk around on eggshells because you do not want to possibly upset them in anyway. It is just that you are walking on eggshells 24 h a day.” (Woodgate et al. 2008, p. 1079). Extra effort was required in new or potentially problematic situations: “I can’t go blindly into any situation … You really have to kind of do a quick overview of the situation knowing what’s going to bother whom.” (Larson 2010, p. 23).

### Managing Non-Compliance with Everyday Tasks and Activities

Parents reported intervening to assist the child with daily activities, such as dressing (e.g., Clarke et al. 1999; Blair et al. 2011): “David would often lie on his bed looking at the ceiling or wall while holding the clothing in his hand until his mother came in to help him. His mother reported that David was physically capable of dressing himself and would often do so quickly before going to a preferred location such as a park.” (Bailey and Blair 2015, p. 224). Parental intervention reduced performance demands and thus decreased the risk of frustration-related problem behaviour: “Her parents reported that as a result of Katherine’s problem behaviors, they had to do everything for her, including dress her, feed her, and help her complete daily hygiene tasks.” (Lucyshyn et al. 2007, p. 133).

Parents also gave the child repeated cues to do things, including verbal reminders and physical prompts (e.g., Hampshire et al. 2016; Neely-Barnes et al. 2011). They used strategies when making demands to reduce the likelihood of non-compliance, e.g., linking activities to the child’s special interests (Larson 2006), tricking the child (Larson 2006; Cullen and Barlow 2002), or giving choices (Larson 2006; Johnson et al. 2014). Gentle persuasion was also used to coax the child into doing things (e.g., “David, a father of a 10-year-old girl with autism, explained how his child would sit down and refuse to be moved: ‘‘Sometimes I have to wait it out and try to coax her.””) (Neely-Barnes et al. 2011, p. 214).

Parents reported using reward systems (e.g., positive behaviour charts), and bargaining to motivate good behaviour and compliance with daily activities (e.g., Bagatell 2015; Dunlap et al. 1994; Fong 1993): “He has to take two bites of a non-preferred food and then reward him with a preferred food. So you know, it’s not like the most relaxing dinner” (Schaaf et al. 2011, p. 381). They also praised the child for appropriate behaviour (Agazzi et al. 2013; Armstrong and Kimonis 2013).

Parents reported persisting with routine demands despite the child’s protests, using a variety of strategies: “Michael would often kick and scream when asked to comply with morning activities […]. The family would continue to deliver verbal demands to comply with activities and would try to ‘‘get him out of the bad mood’’ by tickling or chasing, eventually reverting to yelling, holding him down if he was kicking excessively, or leaving him alone and trying again a few minutes later.” (Sears et al. 2013, p. 1010).

### Responding to Problem Behaviour

Parents reported that distracting the child with activities could divert them from problem behaviour (e.g., Cullen and Barlow 2002; Fettig et al. 2015), and pre-empt outbursts in challenging situations (e.g., Sears et al. 2013; Weiss et al. 2014). Distraction often involved specific activities or items: “[…] each family had developed their own “must have” items. For all the families, technology played a key role. Phones, tablets, and other hand-held devices were common items for families to have charged and ready for an outing.” (Bagatell 2015, p. 55). Children could also be given a task or responsibility (e.g., pushing the trolley in a shop, Schaaf et al. 2011). At home, families put videos on for the child to pre-empt problems and give family members some free time (e.g., DeGrace 2004; Marquenie et al. 2011).

When the child became difficult, parents reported attempting to ignore their demands (e.g., saying “No”, ignoring requests to go to particular places) (e.g., Bailey and Blair 2015; Marquenie et al. 2011), and avoided drawing attention to difficult/inappropriate behaviour in public (e.g., Neely-Barnes et al. 2011; Gray 1993).

Parents reported explicitly teaching the child what is appropriate behaviour, providing verbal explanations and social stories (Armstrong and Kimonis 2013; Ökcün and Akçin 2012; Beer et al. 2013). They gave verbal reprimands in response to problem behaviour (e.g., saying “Don’t”, “Stop”) (e.g., Blair et al. 2011; Dunlap et al. 1994; Gray 1993; Johnson and Whitman 1978). They also established boundaries by setting ground rules in potentially difficult situations (e.g., when going into a shop where the child might want to buy things; e.g., Ryan 2010), and gave punishments by removing items or privileges (e.g., Moes and Frea 2000; Hebert 2014; Armstrong and Kimonis 2013).

Parents shouted, yelled and conveyed negative affect in response to aggression or problem behaviour (e.g., Barry and Singer 2001; Vaughn et al. 2002; Bailey and Blair 2015). Time-out was also used (e.g., Agazzi et al. 2013; Armstrong and Kimonis 2013; Blair et al. 2011), though this could prove challenging: “They had tried using time-out but felt that it was totally ineffective with Carrie because she would yell or leave the time-out area.” (Armstrong et al. 2015, p. 7). Parents also reported using physical punishment in response to problem behaviour: “Her father voiced that he came from “old-school” parenting, but had found that spanking and other forms of punishment such as removing items or privileges, had little effect on Carrie’s behaviour.” (Armstrong et al. 2015, p. 7).

### Managing Distress

When managing extreme distress (e.g., outbursts, meltdowns), parents attempted to comfort the child by providing additional sensory activities, verbal attention (e.g., telling the child “it’s ok” or asking “what’s wrong?”), or physical attention (e.g., hugs, holding or caressing the child) (e.g., Schaaf et al. 2011; Becker-Cottrill et al. 2003; Bourke-Taylor et al. 2010). They also reported removing the child from the situation (Fletcher et al. 2012; Nadeau et al. 2015), or instructing others to leave the vicinity (Flood and Luiselli 2016) to reduce distress and problem behaviour.

### Maintaining Safety

Parents made efforts to physically contain the child to prevent dangerous or destructive behaviour or elopement. This included keeping doors locked and installing motion detectors or other security features so that the child couldn’t leave the house unnoticed (e.g., Myers et al. 2009; Bourke-Taylor et al. 2010; Hutton and Caron 2005; DeGrace et al. 2014). Parents kept the child in a different room away from his/her siblings to mitigate the risk of aggression and injury to siblings (e.g., Gray 1997, 2003; Hodgetts et al. 2013b). They restricted access to valued possessions (particularly those belonging to siblings), or potentially dangerous items (e.g., sharp objects) by installing locks on cupboards and doors (e.g., Hutton and Caron 2005; Bourke-Taylor et al. 2010).

Physical restraint was used to manage aggressive or dangerous outbursts (e.g., Preece 2014): “To actually physically restrain him and not get head butted [is difficult], because, you know, you’ve got his arms, your lying on top of him and he wets his pants and I tell you, it’s a real traumatic experience” (Gray 1997, p. 1104). Restraint was also used to prevent injury to siblings, stop the child running off (e.g. Fairthorne et al. 2014), or curtail dangerous behaviour: “Leonard would engage in problem behavior, such as hitting and kicking his mother or the car, throwing objects at his mother, yelling, hanging out the car window, and not wearing his seat belt appropriately if at all, which often compromised the safety of himself and his mother while driving [...] His mother would respond by yelling at him and physically blocking him or putting him back in his seat” (Bailey and Blair 2015, p. 224).

### Analysing and Planning

Parents reported thinking about what brought on an episode of problem behaviour to develop a more strategic response: “Now there is structure [but I] show a greater…respect for his individuality…I’m not, this is the way we do it…because that doesn’t work. My kid is screaming right back at me so it’s not working… Next time see [what] happens. It goes different… I’m going to be a smooth operator. Slide in there and…study the situation.” (Larson 2006, p. 72).

Parents also tried to anticipate problems that the child might have in a situation (e.g., Fletcher et al. 2012; Lasser and Corley 2008). This was considered essential in successfully negotiating outings: “I have to [be] two steps ahead of him every waking moment when I’m not here in this house… I have to plan ahead every step of the way… There is always going to be a meltdown, something he doesn’t want to do” (Schaaf et al. 2011, p. 383). Preparations for outings or events in the community involved making contingency plans: “I knew Kyle was a little iffy so I told my husband that we should sit on the bleacher on the end so that we wouldn’t have to crawl over people in case we needed to leave early. And we did. I took Kyle home and my husband stayed at the game and watched Kelton play.” (Bagatell 2015, p. 55). Having a plan for any eventuality and changing plans immediately if required allowed families to negotiate outings and manage the risk of problem behaviour (e.g., Marshall and Long 2010; Pepperell et al. 2016; Bagatell 2015; Lutz et al. 2012).

## Discussion

The present study provides novel insight into everyday parenting approaches in response to several important domains of problem behaviour in ASD: irritability, non-compliance, challenging behaviour and anxiety. The meta-synthesis identified numerous descriptions of parenting strategies from the extant literature, which were first summarised descriptively, and then organised into broader concepts. In total, nine higher order concepts were identified: (1) Accommodating the child; (2) modifying the environment; (3) providing structure, routine and occupation; (4) supervision and monitoring; (5) managing non-compliance with everyday tasks and activities; (6) responding to problem behaviour; (7) managing distress; (8) maintaining safety and (9) analysing and planning.

A key finding of this synthesis is the significant complexity of parenting strategies to manage and pre-empt problem behaviour, and the unrelenting burden that meeting the child’s requirements presents. These data imply quantitatively greater and qualitatively more complex parenting demands and parental accommodation in relation to ASD than that which routinely occurs in other populations. This is evidenced by differences between the present descriptions and the dimensions typically studied in parenting research in non-ASD populations, such as positive parenting, involvement, supervision and monitoring, consistency, and discipline (e.g., Essau et al. 2006).

Particularly striking in these data is the extent to which parents manage the child’s propensity for outbursts or problem behaviour by adapting situations, demands, requirements etc. to suit the child, and avoiding direct challenge. As noted by Lucyshyn et al. (2004), this reportedly reflects a decision by parents to ‘preserve the family unit’, where making concessions is perceived to be the lesser evil. This suggests a need for considerably more intervention and support to promote compliance and reduce difficult behaviour than that which appears to be routinely available to parents.

Notably, other parenting strategies reported in this synthesis include more traditional behavioural management approaches, such as consequences, time-out, and physical punishment; suggesting that some parents adopt a far more directive approach to managing problem behaviour. The variation in parental approaches highlights the need for further systematic exploration of how specific child profiles and family factors promote the use of particular strategies.

Studies that assess parenting in the context of child psychopathology often assign a subjective value to particular parenting strategies (e.g., ‘Lax’ parenting), based on theoretical models of the drivers of problem behaviour. Given that problem behaviour in ASD appears to have partially distinct drivers related to specific cognitive vulnerabilities (e.g., poor social awareness; sensory sensitivities; rigidity; heightened anxiety/ emotional dysregulation), optimal parenting strategies in ASD may differ from other populations. Research is needed to systematically explore which parenting strategies are associated with improvements in problem behaviour over time. In addition, work is needed to examine whether different approaches are required for children for whom specific patterns of cognitive drivers appear to trigger problem behaviour.

A strength of this study is its broad focus. We included studies spanning multiple dimensions of problem behaviour in ASD, and a range of ages and clinical profiles. Limitations include the lack of cultural diversity. Study participants were predominantly well-educated Caucasian families living in developed western societies (see Supplementary Tables). In other cultures, where parenting behaviours differ at population levels, strategies to manage problem behaviour in ASD could be quite different.

A further limitation was that studies using systematically measured parenting behaviours were not included in our synthesis, given that we aimed to develop a structure independent of a pre-determined conceptual framework, and embedded within natural contexts. This likely restricted the scope of our findings. In addition, although we aimed to study naturalistic parenting strategies, parents were likely to have been influenced by previous experiences of attending parent training, or advice given to them on managing problem behaviour in ASD. Studies rarely provided information on whether included parents had previously received support or training, so it is impossible to estimate the possible impact of this on our results.

A further consideration is that we cannot be sure, based on the present work, whether the strategies described are likely to be specific to parents of children with ASD as opposed to other neuro-developmental profiles. We included a minority of studies where a subset of individuals did not have an ASD diagnosis, but reportedly presented a similar behavioural challenge. A minority of studies also included reports on strategies from parents of children aged over 18 years. Further work is needed to explore the impact of age and ASD symptomatology on parenting strategies.

Whilst the present work focused on children with ASD, many of these themes would also be recognisable by parents of children with severe intellectual disability. Because exemplars were drawn predominantly from qualitative studies, it was not possible to link them to particular individuals and thus explore the impact of intellectual disability in these data. It remains possible that exemplars disproportionately represent strategies adopted by parents of children with intellectual disability. As such, further work is needed to address their relevance across the full range of ability level.

A further limitation of the study is that only one author (EO) screened the papers against the study inclusion criteria, and identified exemplars from full-texts. In the absence of reliability data, we cannot assess the likelihood that relevant studies were missed. However, the aim of a meta-synthesis is to generate purposive rather than exhaustive sampling, to identify sufficient information to facilitate thematic saturation (Doyle 2003). As such, the omission of relevant material does not compromise the present findings.

This meta-synthesis drew predominantly on qualitative studies, in which exemplars are drawn from specific participants. In this context, exemplars were likely to come from those presenting the most severe behavioural challenges. As such, the use of these strategies for milder manifestations of problem behaviour in ASD remains in question. Research using tailored quantitative methods is now needed to examine the ubiquity of these parenting strategies across the spectrum of problem behaviour severity in ASD.

The majority of existing studies investigating parenting strategies in ASD have used off-the-peg measures formulated with broader clinical populations in mind (e.g., Shawler and Sullivan 2015). This analysis shows that many of the strategies used by parents of children with ASD are specifically targeted to manage particular vulnerabilities (e.g., sensory sensitivities, rigidity, insistence on sameness), or accomplish particular behavioural goals, and may be relatively unique to this population. The present results will be used to inform the development of a questionnaire to measure everyday strategies relevant to the management of problem behaviour in ASD. Researchers interested in following the development and validation of this measure are encouraged to contact the study authors.

## Electronic supplementary material

Below is the link to the electronic supplementary material.

Supplementary material 1 (PDF 285 KB)
