# Supplementary material for: How do Parents Manage Irritability, Challenging Behaviour, Non-Compliance and Anxiety in Children with Autism Spectrum Disorders? A Meta-Synthesis
Source: J Autism Dev Disord. 2017 Dec 8;48(4):1272–86. doi: 10.1007/s10803-017-3361-4 (PMC5861158; doi:10.1007/s10803-017-3361-4)
Supplement: Supplementary file 1 — Supplementary material 1 (PDF 285 KB) [file 10803_2017_3361_MOESM1_ESM.pdf]

Supplementary Materials for ***"How do parents manage irritability, challenging behaviour, non-compliance and anxiety in children with autism spectrum disorders? A meta-synthesis"***

Elizabeth O'Nions, Francesca Happé, Kris Evers, Hannah Boonen & Ilse Noens

Contents:

Supplementary Table 1: Demographic information for case studies/ descriptions (n = 15)

Supplementary Table 2: Demographic information for case series (n = 8)

Supplementary Table 3: Demographic information for qualitative studies (n = 50)

Supplementary Table 4: Summary of themes and references for exemplars coded as belonging to each subtheme

Supplementary Table 1: Demographic information for case studies/ descriptions (n = 15)

Author	Study focus/ location	Sample	Ethnicity (parent or child)	Proportion of parents who were mothers	Parent educational qualifications	Age of child (years)	Child gender	Child intellectual disability and/or delay relative to peers and/or non-verbal/ limited speech
**Agazzi et al. 2013**	Parent-child interaction therapy (US)	Parents of child diagnosed with ID, ASD, ODD, stereotypic movement disorder, primary insomnia	Not given	1/2	Not given	7	M	Yes (diagnosis of ID)
**Armstrong et al. 2013**	Parent-child interaction therapy (US)	Parents of a child diagnosed with Asperger's, ADHD, ODD, OCD	Not given	1/2	Not given	5	M	No (IQ in superior range)
**Armstrong et al. 2015**	Parent-child interaction therapy with visual supports (US)	Parents of a child diagnosed with epilepsy, ASD, ID and ADHD	Not given	1/2	Not given	5	F	Yes (diagnosis of ID)
**Barry and Singer 2001**	Clinician-implemented treatment (US)	Parents of a child diagnosed with limited speech, autism, aggressive behaviour	Not given	1/2	Not given	10	M	Yes (limited speech)
**Becker-Cottrill et al. 2003**	Implementation of multi-component intervention (US)	Parents of a child diagnosed with autism	Not given	1/2	Not given	4	M	Yes (limited speech)
**Clarke et al. 1999**	Family-centred assessment-based intervention (US)	Parents of a child diagnosed with Asperger's, non-compliance and tantrums	Not given	1/2	Not given	10	M	No (Asperger's diagnosis)

Author	Study focus/ location	Sample	Ethnicity (parent or child)	Proportion of parents who were mothers	Parent educational qualifications	Age of child (years)	Child gender	Child intellectual disability and/or delay relative to peers and/or non-verbal/ limited speech
**Flood and Luiselli 2016**	Desensitization treatment (US)	Parents of a child diagnosed with autism	Not given	1/2	Not given	7	M	Yes (verbal but limited expressive language)
**Johnson et al. 1978**	Home-based therapeutic intervention (US)	Parents of a child diagnosed with autism	Not given	1/2	Well-educated	4	F	Not given
**Lucyshyn et al. 2007**	Positive behaviour support approach (US)	Parents of a child diagnosed with severe ID, autism, challenging behaviour	Not given	1/2	Not given	5	F	Yes (diagnosis of ID)
**Moes and Frea 2000**	Prescriptive vs. contextualized treatment approach (US)	Parents of a child diagnosed with autism, mood disorder	Not given	1/2	Not given	3	M	No (age appropriate cognitive abilities)
**Nadeau et al. 2015**	Behaviourally oriented psychosocial intervention (US)	Parents of a child diagnosed with autism, GAD, social phobia	Hispanic	1/2	Not given	4	M	Yes (minimal expressive language)
**Neufeld et al. 2014**	Effectiveness of multi-component intervention (Canada)	Mother of a child diagnosed with autism and anxiety problems	Not given	1/1	Not given	5	M	No (verbal, spoke in full sentences)
**Ökcün et al. 2012**	Parent behaviours to support play with peers (Turkey)	Mother of a child diagnosed with autism	Not given	1/1	Not given	9	M	Not given

**Author**	**Study focus/ location**	**Sample**	**Ethnicity (parent or child)**	**Proportion of parents who were mothers**	**Parent educational qualifications**	**Age of child (years)**	**Child gender**	**Child intellectual disability and/or delay relative to peers and/or non-verbal/ limited speech**
**Sabapathy et al. 2016**	Aggressive behaviour (US)	Parents of a child diagnosed with ASD, seizure disorder, moderate ID and aggressive behaviour	Hispanic	1/2	Not given	13	F	Yes (diagnosis of ID)
**Vaughn et al. 2002**	Family-centred intervention (US)	Parents of child diagnosed with severe ID, autism, challenging behaviour	African-American	1/2	Not given	7	M	Yes (diagnosis of ID)

Notes: Abbreviations: US = United States of America; ASD = Autism spectrum disorder; ID = intellectual disability; GAD = Generalised Anxiety Disorder; ODD = Oppositional Defiant Disorder; OCD = Obsessive compulsive disorder; M = Male; F = Female.

Supplementary Table 2: Demographic information for case series (n = 8)

Author	Study focus/ location	Sample	Ethnicity (parent or child)	Proportion of parents who were mothers	Parent educational qualifications	Age of children [years] mean (range)	Proportion of children who were female	Proportion of children with intellectual disability (ID) and/or delay relative to peers and/or non-verbal/ limited speech
**Bailey and Blair 2015**	Family-centred Prevent-Teach-Reinforce (US)	Parents of children diagnosed with ASD or language delay, sensory processing problems, problem behaviour [a]	Caucasian	3/6	Not given	6 (5 - 7)	0/3	3/3 (limited speech)
**Blair et al. 2011**	Individualised behaviour support (South Korea)	Parents of children diagnosed with ID, autism, challenging behaviour, non-compliance, self-injury, cerebral palsy (1/3)	Not given	3/3	33% High school diploma; 67% College degrees	4.8 (4 - 5)	1/3	3/3 (diagnosis of ID)
**Fettig et al. 2015**	Coaching to support parenting intervention (US)	Parents of children diagnosed with autism or sensory integration disorder and challenging behaviour/ poor compliance [a]	Not given	3/6	Not given	4.4 (2 - 6)	1/3	1/3 (cognitive delays)
**Hampshire et al. 2011**	Parent training, self-management and homework independence (US)	Parents of children with ASD who experienced homework challenges	Not given	5/5	Not given	(11 - 14)	1/5	Not given
**Lucyshyn et al. 2004**	Coercive cycles in unsuccessful family routines (US)	Parents of children with ASD, delayed language skills or mild to moderate mental retardation [a]	Not given	10/12	Not given	(4 - 8)	Not given	6/10 (non-verbal or delayed language)

Author	Study focus/ location	Sample	Ethnicity (parent or child)	Proportion of parents who were mothers	Parent educational qualifications	Age of children [years] mean (range)	Proportion of children who were female	Proportion of children with intellectual disability (ID) and/or delay relative to peers and/or non-verbal/ limited speech
**Preece and Almond 2008**	Structured teaching approaches (UK)	Parents of children on the autism spectrum	Not given	2/3	Not given	8.5 (6 – 11)	1/2	Not given
**Scarpinto et al. 2010**	Management of behaviour in hospital setting (US)	Health-care providers of hospitalised children with ASD	Not given	Not given (focus is health-care providers, also reported parent behaviours)	Not given	(6 - 15)	1/9	Not given
**Sears et al. 2013**	School-based Prevent-Teach-Reinforce intervention (US)	Parents of children diagnosed with ASD with problem behaviour	Caucasian	2/4	Not given	5 (4 - 6)	0/2	1/2 (non-verbal)

Abbreviations: US = United States of America; UK = United Kingdom; ASD = Autism spectrum disorder; PDD-NOS = Pervasive developmental disorder not otherwise specified; ID = intellectual disability; GAD = Generalised Anxiety Disorder; ODD = Oppositional Defiant Disorder; OCD = Obsessive compulsive disorder. Notes: [a] study includes a minority of children without an ASD.

Supplementary Table 3: Demographic information for qualitative studies (n = 50)

Author	Study focus/ location	Sample	Ethnicity (parent or child)	Proportion of parents who were mothers	Parent educational qualifications	Age of children [years] mean (range)	Proportion of children who were female	Proportion of children with intellectual disability (ID) and/or delay relative to peers and/or non-verbal/ limited speech
**Ausderau and Juarez 2013**	Eating challenges and mealtimes (US)	Parents of children with ASD and feeding challenges	83% Caucasian, 17% Hispanic	6/6	17% High School; 67% College; 17% Advanced	4.7 (2 - 7)	Not given	Not given
**Aylaz et al. 2012**	Parents' difficulties, impact on their sex life (Turkey)	Parents of children with autism	Not provided	6/12	42% High school; 33% University; 25% Primary school	10 (6 - 14)	Not given	Not given
**Bagatell 2016**	Family routines and occupations (US)	Parents and siblings of children with moderate – severe ASD [b]	60% Caucasian; 20% Hispanic; 20% African American	5/6	20% High school; 80% College	14.8 (13 – 18)	0/5	Not given
**Hebert 2014**	Factors affecting choice of intervention (US)	Parents of children with PDD-NOS or autism	74% Caucasian; 17% African American; 9% Asian	14/23	17% High School; 22% College; 60% Advanced	4.5 (3 - 6)	7/19	14/19 (diagnosed with autism rather than PDD-NOS or Asperger's)
**Bearss et al. 2016**	Anxiety behaviours and management (US)	Parents of children with ASD and anxiety	67% Caucasian, 4% Hispanic, 18% African American, 11% other	41/48	27% Some college, 42% College graduate, 31% Advanced degree	10.4 (3 - 17)	13/45	21/45 (diagnosed with autism rather than PDD-NOS or Asperger's)
**Beer et al. 2013**	Mindful parenting (Australia)	Parents of children with ASD	87% Caucasian	24/28	33% High school; 67% College or Advanced	9.0 (3 - 20)	85.7% male (child N not given)	Not given

Author	Study focus/ location	Sample	Ethnicity (parent or child)	Proportion of parents who were mothers	Parent educational qualifications	Age of children [years] mean (range)	Proportion of children who were female	Proportion of children with intellectual disability (ID) and/or delay relative to peers and/or non-verbal/ limited speech
**Bourke-Taylor et al. 2010**	Family impact: mothers' perspectives (Australia)	Mothers of children with autism or cerebral palsy [c]	Not provided	2/2	Not provided	12 (9 - 15)	1/2	1/2 (diagnosis of ID)
**Cullen and Barlow 2002**	Caring for children attending touch therapy (UK)	Parents of children with autism	90% Caucasian; 10% Asian	9/10	Not provided	(2 - 13)	1/10	6/10 (non-verbal/ limited speech)
**DeGrace et al. 2014**	Lived experience: daily life (US)	Parents of children with severe autism	Not provided	7/13	Not provided	12.4 (3 - 18)	Not given	Not given
**DeGrace 2004**	Lived experience: daily life (US)	Parents and siblings of children with autism [b]	Not provided	5/10	Not provided	9.8 (9 - 10)	2/5	Not given
**Dickie et al. 2009**	Sensory experiences (US)	Parents of children with or without autism (child data refers to autism sub-sample) [c]	73% Caucasian; 16 % Black; 5% Hispanic; 5% Mixed	37/37	27% High school; 16% Partial college; 41% College degree; 3% Beyond college; 16% Graduate degree	4.43 (2-7)	Not given	Not given
**Divan et al. 2012**	Challenges, coping strategies and unmet needs of families (India)	Parents and stakeholders of children with ASD [c]	Not provided	9/12	10% School; 20% High school; 70% Graduate	Not provided, includes under 5 and above 20, majority under 18	1/10	Not given

Author	Study focus/ location	Sample	Ethnicity (parent or child)	Proportion of parents who were mothers	Parent educational qualifications	Age of children [years] mean (range)	Proportion of children who were female	Proportion of children with intellectual disability (ID) and/or delay relative to peers and/or non-verbal/ limited speech
**Duignan and Connell 2015**	Lived experience with ASD (Australia)	Parents/ carers and adult siblings of children with ASD [b]	Not provided	26/29	Not provided	(2 - 57)	Not given	Not given
**Dunlap et al. 1994**	Challenging behaviour and strategies (US)	Parents of children with autism or autistic features [a]	Not provided	72/78	Not provided	11.6 (2 - 38)	17/79	Not given
**Fairthorne et al. 2014**	Experiences impacting mothers' quality of life (Australia)	Mothers of children with autism and ID	Not provided	16/16	Not provided	15.6 (11 - 24)	6/17	17/17 (reported to have ID)
**Farrugia 2009**	Experience of stigma (Australia)	Parents of children with ASD	Anglo-Saxon	11/16	Not provided	(5 - 23)	Not given	Not given
**Fletcher et al. 2012**	Perceived costs of caring (Canada)	Mothers of children with ASD	Not provided	8/8	All completed high school or college/ university	Not given, maximum age 12	Not given	Not given
**Fong et al. 1993**	Experience of parenting (Canada)	Parents of children with autism	Not provided	6/8	All had at least Grade 12 education, several with post-secondary or professional education	14.2 (13 - 15)	1/6	3/6 (behind age peers academically)

Author	Study focus/ location	Sample	Ethnicity (parent or child)	Proportion of parents who were mothers	Parent educational qualifications	Age of children [years] mean (range)	Proportion of children who were female	Proportion of children with intellectual disability (ID) and/or delay relative to peers and/or non-verbal/ limited speech
**Foo et al. 2015**	Lived experience with autism and challenging behaviour (Singapore)	Parents of children with ASD and aggression/ challenging behaviour	83% Chinese, 17% Indian	3/6	16% High school; 33% College; 50% Advanced	12.8 (10 - 18)	2/6	5/6 (diagnosed with autism rather than PDD-NOS or Asperger's)
**Gray 1993**	Perceptions of stigma (Australia)	Parents of children with autism	Not provided	23/32	Modal value: high school	Not provided, includes under age 12	Not given	Not given
**Gray 1997**	The construction of "normal family life" (Australia)	Parents of children with high functioning autism or Asperger's syndrome	Not provided	32/53	Not provided	(5 - 26), majority aged 9 – 14	Not given	Child N not given, all reported to have high-functioning autism or Asperger's
**Gray 2003**	Gender and parental coping (Australia)	Parents of children with high functioning autism or Asperger's syndrome	Not provided	32/53	Not provided	(5-26), majority aged 9 – 14	Not given	Child N not given, all reported to have high-functioning autism or Asperger's
**Hodgetts et al., 2013a**	Experience/ outcomes of intervention (Canada)	Parents of children with ASD	60% Caucasian; 20% Middle Eastern; 20% Southeast Asian	10/15	Not provided	8.2 (5 - 12)	2/10	5/10 (report of very low adaptive functioning/ cognition untestable)
**Hodgetts et al. 2013b**	Experiences with aggression (Canada)	Parents of children with ASD	100% Caucasian	8/9	All had at least a high school diploma	(6 - 29), majority aged 6 - 10	0/9	5/9 (moderate to severe ID reported by parents)
**Hutton and Caron 2005**	Lived experience (US)	Parents of children with autism	Not provided	19/21	Not provided	(3-16)	4/21	Not given

Author	Study focus/ location	Sample	Ethnicity (parent or child)	Proportion of parents who were mothers	Parent educational qualifications	Age of children [years] mean (range)	Proportion of children who were female	Proportion of children with intellectual disability (ID) and/or delay relative to peers and/or non-verbal/ limited speech
**Johnson et al. 2014**	Challenging behaviour in hospitalised children (US)	Mothers and health-care providers of children with ASD [c]	80% Caucasian, 20% African American	5/5	20% High school; 40% College; 40% Advanced	(5 - 12)	Not given	Not given
**Koydemir-Özden and Tosun 2010**	Lived experience: Turkish mothers (Turkey)	Mothers of children with autism	Not provided	13/13	Not provided	(7-14)	Not given	Not given
**Kuhaneck et al. 2010**	Maternal coping (US)	Mothers of children with ASD	82% Caucasian; 18% African American	11/11	Not provided	8.3 (6-11)	Not given	Not given
**Larson 2006**	Children's propensity for routinization (US)	Mothers of children with ASD plus an additional difficulty (e.g. non-verbal, problem behaviour)	Varied	9/9	Not provided	8.7 (3 - 14)	0/10	6/10 (diagnosed with autism rather than PDD-NOS or Asperger's)
**Larson 2010**	Maternal support of participation in everyday life (US)	Parents of children with ASD	67% Caucasian, 11% Mixed, 11% Chinese, 11% Hispanic	9/9	Not provided	(3 - 14)	0/10	1/10 (the rest reported by parents to be high-functioning)
**Lasser and Corley 2008**	Parenting (US)	Parents of children with Asperger's disorder	Not provided	19/20	10% High school; 40% College; 40% Graduate school; 10% not reported	11.98	3/21	0/21 (all had a diagnosis of Asperger's)

Author	Study focus/ location	Sample	Ethnicity (parent or child)	Proportion of parents who were mothers	Parent educational qualifications	Age of children [years] mean (range)	Proportion of children who were female	Proportion of children with intellectual disability (ID) and/or delay relative to peers and/or non-verbal/ limited speech
**Ludlow et al. 2012**	Challenges faced by parents of children with ASD (UK)	Parents of children with ASD	Not provided	14/20	Not provided	(4 - 29), majority aged 4 - 14	Not given	8/14 (diagnosed with autism rather than PDD-NOS or Asperger's)
**Lutz et al. 2012**	Familial coping/adaptatio n (US)	Mothers of children with autism	88% Caucasian, 12% African American	16/16	Not provided	(2 - 31), majority under 18	1/16	16/16 (diagnosed with "classic autism")
**Marquenie et al. 2011**	Dinner and bedtimes routines and rituals (Australia)	Mothers of children with ASD	Not provided	14/14	21% High school; 14% College; 57% Advanced; 7% Technical education	3.4 (3 - 4)	Not given	Not given
**Marshall and Long 2010**	Maternal coping processes (Canada)	Mothers of children with autism	100% Caucasian	5/5	40% Secondary school; 60% University	(6-11)	Not given	Not given
**Mount and Dillon 2014**	Lived experience (UK)	Parents of children with ASD	100% Caucasian	5/9	Not provided	13 (11 - 16)	1/6	Not given
**Myers et al. 2009**	Lived experience: impact on parents' lives (US)	Parents of children with ASD	87% Caucasian, 6% Mixed, 2% African American, 2% Hispanic, 3% other	455/493	14% High school; 41% College; 37% Advanced	8.6 (1 - 21)	99/493	295/493 (diagnosed with autism rather than PDD-NOS or Asperger's)
**Neely-Barnes et al. 2011**	Parenting & public perceptions (US)	Parents of children with ASD	46% Caucasian; 36% African American; 9% Asian American; 9% Latina	9/11	37% High school; 27% Some college; 9% College degree; 27% Graduate	7.2 (1-15)	5/14	Not given

Author	Study focus/ location	Sample	Ethnicity (parent or child)	Proportion of parents who were mothers	Parent educational qualifications	Age of children [years] mean (range)	Proportion of children who were female	Proportion of children with intellectual disability (ID) and/or delay relative to peers and/or non-verbal/ limited speech
**Pengelly et al. 2009**	Home environment (UK)	Mothers of children with ASD	Not provided	3/3	Not provided	(8 - 10)	0/3	Not given
**Pepperell et al. 2016**	Parents' social support and coping strategies (Australia)	Parents of children with ASD	Not provided	10/19	26% High school; 32% College/vocational; 42% Advanced	4.1 (3 - 4)	4/10	Not given
**Preece 2014**	Follow up of behavioural support and physical intervention training (UK)	Parents of children with ASD or ADHD and challenging behaviour [a]	Not provided	Not given	Not provided	9.7 (7 - 11)	Not given	Not given
**Ryan 2010**	Going out in the community (UK)	Parents of children with ASD	Mostly Caucasian	36/48	Not provided	(3 - 53)	Not given	Not given
**Safe et al. 2012**	Lived experience: mothers (Australia)	Mothers of children with autism	86% Caucasian; 14% Asian	7/7	14% Certificate; 43% Year 12; 43% Degree	(6-12)	2/9	9/9 (diagnosed with autism rather than PDD-NOS or Asperger's)
**Schaaf et al. 2011**	Everyday routines (US)	Parents of children with ASD and sensory difficulties	100% Caucasian	4/5	100% College degree	8.25 (7-12)	Not given	Not given
**Shaked 2005**	Lived experience: ultraorthodox community (Israel)	Parents of children assessed as autistic (18/30 had a formal diagnosis of an ASD) [a]	Not provided	29/32	Not provided	Not provided, includes children aged under 18	Not given	16/30 (diagnosed with autism rather than PDD-NOS or Asperger's)

Author	Study focus/ location	Sample	Ethnicity (parent or child)	Proportion of parents who were mothers	Parent educational qualifications	Age of children [years] mean (range)	Proportion of children who were female	Proportion of children with intellectual disability (ID) and/or delay relative to peers and/or non-verbal/ limited speech
**Stoner et al. 2007**	Parent perspectives on transitions (US)	Parents of children with ASD	Not provided	4/8	Not provided	7 (6 - 8)	0/4	3/4 (non-verbal/ limited speech)
**Turnbull and Ruef 1996**	Family perspectives on problem behaviour (US)	Parents of children with ID and problem behaviour, (almost all reported to have autism) [a]	76% Caucasian, 24% Latino	15/20	Broad range	14 (2 - 36)	Not given	17/17 (Diagnosis of ID)
**Weiss et al. 2014**	Experience of crisis (Canada)	Mothers of children with ASD	89% Caucasian	155/155	Not provided	15.3 (5 - 48)	28/155	78/155 (diagnosed with autism rather than PDD-NOS or Asperger's)
**Woodgate et al. 2008**	Lived experience (Canada)	Parents of children with autism	90% Caucasian; 10% Asian	16/21	Not provided	(3 - 9)	1/16	Not given, sample reportedly varied in severity
**Zhou and Yi 2014**	Parenting styles & emotions: impact on child functioning (China)	Parents of children with ASD	Not provided	25/32	13% High school; 0% College; 87% Advanced	6.8 (2 - 17)	1/28	Not given

Abbreviations: US = United States of America; UK = United Kingdom; ASD = Autism spectrum disorder; PDD-NOS = Pervasive developmental disorder not otherwise specified; ID = intellectual disability; GAD = Generalised Anxiety Disorder; ODD = Oppositional Defiant Disorder; OCD = Obsessive compulsive disorder. Notes: [a] study includes a minority of children without an ASD; [b] study includes a minority of other relatives or carers (e.g. grandparents, siblings); [c] study includes a larger proportion not diagnosed with ASD or another non-parent informant group, but exemplars are drawn from/about ASD sample and/or parents only.

Supplementary Table 4: Summary of themes and references for exemplars coded as belonging to each subtheme

	Sub-theme	References for exemplars coded as belonging to each subtheme
	(1) Accommodating the child	
1	Adapt routines to accommodate the child	Ausderau and Juarez 2013; Bagatell 2016; Clarke et al. 1999; Duignan and Connell 2015; Farrugia 2009; Lucyshyn et al. 2004; Marquenie et al. 2011; Schaaf et al. 2011
2	Plan activities to accommodate the child	Farrugia 2009; Fletcher et al. 2012; Hutton and Carron 2005; Larson 2006; Lucyshyn et al. 2004; Lutz et al. 2012; Mount and Dillon 2014; Schaaf et al. 2011; Sears et al. 2013; Stoner et al. 2007
3	Adjust expectations depending on the child's mood	DeGrace 2004; Fletcher et al. 2012; Foo et al. 2015; Gray 2003; Larson 2006; Safe et al. 2012; Schaaf et al. 2011
4	Set priorities and pick battles	Ausderau and Juarez 2013; DeGrace 2004; Farrugia 2009; Larson 2006; Marquenie et al. 2011; Safe et al. 2012
5	Give the child latitude over rules	Ausderau and Juarez 2013; Bagatell 2016; Herbert 2014; Bearss et al. 2016; DeGrace 2004; Foo et al. 2015; Larson 2006; Marquenie et al. 2011; Pengelly et al. 2009; Turnbull and Ruef 1996
6	Reduce demands when problem behavior occurs	Becker-Cottrill et al. 2003; Clarke et al. 1999; Foo et al. 2015; Johnson et al. 1978; Larson 2006; Lucyshyn et al. 2004; Lucyshyn et al. 2007; Neufeld et al. 2014; Sears et al. 2013
7	Give in to the child's demands	Armstrong et al. 2015; Aylaz et al. 2012; Bagatell 2016; DeGrace 2004; Divan et al. 2012; Duignan and Connell 2015; Fettig et al. 2015; Fletcher et al. 2012; Hodgetts et al. 2013b; Koydemir-Özden and Tosun 2010; Lucyshyn et al. 2004; Lucyshyn et al. 2007
	(2) Modifying the environment	
8	Limit the child's exposure to sensory stimuli they find aversive	Bagatell 2016; Dickie et al. 2009; Duignan and Connell 2015; Larson 2010; Lucyshyn et al. 2004; Ludlow et al. 2012; Mount and Dillon 2014; Schaaf et al. 2011; Sears et al. 2013
9	Avoid situations (e.g. activities, events, places) that the child finds difficult	Ausderau and Juarez 2013; Bagatell 2016; Divan et al. 2012; Fairthorne et al. 2014; Fletcher et al. 2012; Gray 1997; Gray 2003; Johnson et al. 2014; Larson 2006; Mount and Dillon 2014; Ryan 2010; Schaaf et al. 2011
10	Limit social activities and outings with the child	Bagatell 2016; DeGrace et al. 2014; Divan et al. 2012; Duignan and Connell 2015; Gray 2003; Fairthorne et al. 2014; Fletcher et al. 2012; Hodgetts et al. 2013a; Hodgetts et al. 2013b; Larson 2006; Lucyshyn et al. 2007; Mount and Dillon 2014; Myers et al. 2009; Neufeld et al. 2014; Preece and Almond 2008; Ryan 2010; Schaaf et al. 2011

	Sub-theme	References for exemplars coded as belonging to each subtheme
	(3) Providing structure, routine and familiarity	
11	Stick to fixed routines to manage daily activities	Agazzi et al. 2013; Farrugia 2009; Fong et al. 1993; Hutton and Carron 2005; Kuhaneck et al. 2010; Larson 2006; Ludlow et al. 2012; Mount and Dillon 2014; Safe et al. 2012; Schaaf et al. 2011; Turnbull and Ruef 1996
12	Provide structure and occupation for the child at all times	DeGrace 2004; Duignan and Connell 2015; Dunlap et al. 1994; Johnson et al. 2014; Larson 2006; Marquenie et al. 2011; Pepperell et al. 2016; Schaaf et al. 2011; Turnbull and Ruef 1996
13	Use picture schedules or lists to inform the child about upcoming activities	Bagatell 2016; Clarke et al. 1999; Fettig et al. 2015; Johnson et al. 2014; Larson 2010; Scarpinto et al. 2010; Schaaf et al. 2011; Stoner et al. 2007; Turnbull and Ruef 1996
14	Inform the child in advance about any changes in routine	Bagatell 2016; Duignan and Connell 2015; Gray 1997; Larson 2006; Ludlow et al. 2012; Mount and Dillon 2014; Scarpinto et al. 2010
15	Prepare the child for events by giving details in advance	Bearss et al. 2016; Gray 1997; Johnson et al. 2014; Larson 2006; Ludlow et al. 2012; Marshall and Long 2010; Ryan 2010; Schaaf et al. 2011; Sears et al. 2013
16	Keep things as predictable and familiar as possible	Gray 1997; Johnson et al. 2014; Kuhaneck et al. 2010; Larson 2006; Ludlow et al. 2012; Safe et al. 2012; Stoner et al. 2007
17	Introduce new things gradually	Ausderau and Juarez 2013; Fong et al. 1993; Larson 2006; Larson 2010; Marquenie et al. 2011; Schaaf et al. 2011; Stoner et al. 2007
	(4) Supervision and monitoring	
18	Supervise the child at all times	Bourke-Taylor et al. 2010; DeGrace 2004; Duignan and Connell 2015; Fairthorne et al. 2014; Fletcher et al. 2012; Fong et al. 1993; Hampshire et al. 2011; Hodgetts et al. 2013a; Koydemir-Özden and Tosun 2010; Larson 2010; Lutz et al. 2012; Marshall and Long 2010; Myers et al. 2009; Schaaf et al. 2011; Turnbull and Ruef 1996; Weiss et al. 2014; Zhou and Yi 2014
19	Stay alert and ready to intervene	Barry and Singer 2001; Bourke-Taylor et al. 2010; DeGrace et al. 2014; Hodgetts et al. 2013a; Larson 2010; Marshall and Long 2010; Myers et al. 2009; Ryan 2010; Safe et al. 2012; Schaaf et al. 2011; Woodgate et al. 2008
20	Make an effort to keep the child's mood stable	Gray 1997; Larson 2010; Sabapathy et al. 2016; Woodgate et al. 2008
21	Be vigilant to identify possible triggers	Hutton and Carron 2005; Larson 2010; Schaaf et al. 2011

	Sub-theme	References for exemplars coded as belonging to each subtheme
	(5) Managing non-compliance with everyday tasks and activities	
22	Intervene to assist the child with daily activities	Bailey and Blair 2015; Blair et al. 2011; Clarke et al. 1999; Fletcher et al. 2012; Larson 2006; Larson 2010; Lucyshyn et al. 2004; Lucyshyn et al. 2007; Mount and Dillon 2014; Neufeld et al. 2014; Turnbull and Ruef 1996
23	Give the child repeated cues to do things	Armstrong et al. 2015; DeGrace 2004; Fettig et al. 2015; Flood and Luiselli 2016; Hampshire et al. 2011; Larson 2010; Marquenie et al. 2011; Neufeld et al. 2014; Ökcün and Akçin 2012; Sears et al. 2013; Vaughn et al. 2002
24	Use strategies when making demands	Bearss et al. 2016; Johnson et al. 2014; Larson 2006; Cullen and Barlow 2002; Larson 2006
25	Use gentle persuasion to coax the child into doing things	Agazzi et al. 2013; Neely-Barnes et al. 2011
26	Use reward reward systems to motivate good behaviour	Armstrong et al. 2013; Bagatell 2016; Dunlap et al. 1994; Fettig et al. 2015; Fong et al. 1993; Johnson et al. 2014; Schaaf et al. 2011
27	Praise the child for appropriate behaviour	Agazzi et al. 2013; Armstrong et al. 2013
28	Persist with routine demands despite protests	Marquenie et al. 2011; Mount and Dillon 2014; Sears et al. 2013; Stoner et al. 2007
	(6) Responding to problem behaviour	
29	Distract the child with activities	Bagatell 2016; Cullen and Barlow 2002; Duignan and Connell 2015; Fettig et al. 2015; Johnson et al. 2014; Larson 2006; Marshall and Long 2010; Sears et al. 2013; Weiss et al. 2014
30	Ignore the child's demands	Bailey and Blair 2015; Dunlap et al. 1994; Gray 1993; Marquenie et al. 2011; Neely-Barnes et al. 2011
31	Teach the child what is appropriate behaviour	Armstrong et al. 2013; Beer et al. 2013; Dunlap et al. 1994; Johnson et al. 2014; Larson 2006; Ökcün and Akçin 2012; Woodgate et al. 2008
32	Give verbal reprimands in response to problem behaviour	Blair et al. 2011; Dunlap et al. 1994; Gray 1993; Sears et al. 2013
33	Establish boundaries by setting ground rules	Agazzi et al. 2013; Herbert 2014; Johnson et al. 1978; Pengelly et al. 2009; Ryan 2010
34	Give punishments by removing items or privileges	Armstrong et al. 2013; Armstrong et al. 2015; Herbert 2014; Moes and Frea 2000
35	Shout/ yell and convey negative affect in response to problem behaviour	Bailey and Blair 2015; Barry and Singer 2001; Blair et al. 2011; Gray 2003; Sears et al. 2013; Vaughn et al. 2002
36	Put the child in time-out in response to problem behaviour	Armstrong et al. 2013; Armstrong et al. 2015; Blair et al. 2011; Dunlap et al. 1994; Fong et al. 1993

	Sub-theme	References for exemplars coded as belonging to each subtheme
37	Use physical punishment in response to problem behaviour	Armstrong et al. 2015; Fairthorne et al. 2014
	(7) Managing distress	
38	Comfort the child when they are having an outburst	Bearss et al. 2016; Becker-Cottrill et al. 2003; Bourke-Taylor et al. 2010; Flood and Luiselli 2016; Johnson et al. 2014; Lucyshyn et al. 2004; Ludlow et al. 2012; Neely-Barnes et al. 2011; Schaaf et al. 2011; Turnbull and Ruef 1996; Weiss et al. 2014
	(8) Maintaining safety	
39	Physically contain the child	Aylaz et al. 2012; Bourke-Taylor et al. 2010; DeGrace et al. 2014; Fairthorne et al. 2014; Hodgetts et al. 2013a; Hutton and Carron 2005; Myers et al. 2009; Neely-Barnes et al. 2011
40	Keep the child in a different room away from his/her siblings	Gray 1997; Gray 2003; Hodgetts et al. 2013a
41	Restrict access to valued possessions	Bourke-Taylor et al. 2010; Duignan and Connell 2015; Farrugia 2009; Gray 1997; Hodgetts et al. 2013a; Hutton and Carron 2005
42	Physically restrain the child in response to dangerous behaviour	Agazzi et al. 2013; Bailey and Blair 2015; Barry and Singer 2001; Blair et al. 2011; Bourke-Taylor et al. 2010; Gray 1997; Larson 2010; Ludlow et al. 2012; Preece and Almond 2008; Preece 2014; Sears et al. 2013; Weiss et al. 2014
	(9) Analysing and planning	
43	Think about what brought on an episode of problem behaviour	Farrugia 2009; Hieneman and Dunlap 2000; Larson 2006; Larson 2010; Whittingham et al. 2006
44	Anticipate problems that the child might have in a situation	Farrugia 2009; Fletcher et al. 2012; Gray 2003; Larson 2010; Lasser 2008; Safe et al. 2012; Schaaf et al. 2011; Stoner et al. 2007
45	Make contingency plans for outings	Bagatell 2016; Gray 2003; Larson 2006; Marshall and Long 2010; Pepperell et al. 2016; Schaaf et al. 2011
